# Effects of Exogenous Transferrin on the Regulation of Iron Metabolism and Erythropoiesis in Iron Deficiency With or Without Anemia

**DOI:** 10.3389/fphys.2022.893149

**Published:** 2022-05-11

**Authors:** Yihang Li, Ian Miller, Princy Prasad, Nisha Ajit George, Nermi L. Parrow, Robert E. Fleming

**Affiliations:** ^1^ Department of Pediatrics, Saint Louis University School of Medicine, St. Louis, MO, United States; ^2^ Edward A. Doisy Department of Biochemistry and Molecular Biology, Saint Louis University School of Medicine, St. Louis, MO, United States

**Keywords:** transferrin, iron, iron deficiency, erythropoiesis, anemia, hepcidin, erythropoietin, erythroferrone

## Abstract

Erythropoietic response is controlled not only by erythropoietin but also by iron. In addition to its role in iron delivery, transferrin also functions as a signaling molecule, with effects on both iron homeostasis and erythropoiesis. We investigated hematologic parameters, iron status and expression of key proteins, including the hepatic iron regulatory protein hepcidin and the suppressive erythroid factor *Erfe*, in mice subject to dietary iron deficiency with and without anemia. The acute effect of iron on these parameters was investigated by administration of exogenous iron-loaded transferrin (holoTf) in each of the mouse models. Serum iron in mice with iron deficiency (ID) is modestly lower with hematologic parameters maintained by utilization of iron stores in mice with ID. As expected, erythropoietin expression and concentration, along with marrow *Erfe* are unaffected in ID mice. Administration of holoTf restores serum iron and Tf saturation levels to those observed in control mice and results in an increase in hepcidin compared to ID mice not treated with holoTf. The expression of the Bmp signaling molecule *Bmp6* is not significantly increased following Tf treatment in ID mice. Thus, the expression level of the gene encoding hepcidin, *Hamp1*, is increased relative to *Bmp6* expression in ID mice following treatment with holoTf, leading us to speculate that Tf saturation may influence Bmp sensitivity. In mice with iron deficiency anemia (IDA), decreased hematologic parameters were accompanied by pronounced decreases in serum and tissue iron concentrations, and an increase in serum erythropoietin. In the absence of exogenous holoTf, the greater serum erythropoietin was not reflected by an increase in marrow *Erfe* expression. HoloTf administration did not acutely change serum Epo in IDA mice. Marrow *Erfe* expression was, however, markedly increased in IDA mice following holoTf, plausibly accounting for the lack of an increase in *Hamp1* following holoTf treatment in the IDA mice. The increase in *Erfe* despite no change in erythropoietin suggests that Tf acts to increase erythropoietin sensitivity. These observations underscore the importance of Tf in modulating the erythropoietic response in recovery from iron deficiency anemia, with implications for other stress erythropoiesis conditions.

## Introduction

During stress erythropoiesis iron utilization for hemoglobin production increases markedly. As such compensatory mechanisms are essential to ensure an adequate iron supply to meet this need. Transferrin is a likely candidate to communicate iron status from the marrow to the liver. Transferrin has two receptors, TfR1 and TfR2. TfR1 is the primary receptor for transferrin mediated iron delivery, although TfR1-mediated signaling has been reported (Coulon et al., 2011; Kenneth et al., 2013). Several lines of evidence support a role for TfR2 in modulating signaling events controlling iron homeostasis and erythropoiesis. TfR2 is expressed in multiple cell types, including hepatocytes and erythroid precursor cells. Loss or mutation of hepatocellular TfR2 results in dysregulation of the iron regulatory hormone, hepcidin, resulting in hereditary hemochromatosis (Camaschella et al., 2000; Fleming et al., 2002; Wallace et al., 2007). Bone morphogenetic proteins (Bmps), primarily Bmp2 and Bmp6 (Canali et al., 2017), upregulate expression of the hepatic iron regulatory protein hepcidin in response to iron through the SMAD signaling pathway (Kautz et al., 2008) In addition to responding to acute and chronic changes in iron status, hepcidin is suppressed by the erythroid demand and this suppression of hepcidin is an important component of the stress erythropoiesis response. Suppression is mediated, at least in part, by erythroferrone (Erfe) (Kautz et al., 2015), which is produced by erythroblasts in response to erythropoietin (Epo) and appears to interfere with Bmp/Smad signaling (Wang et al., 2020). Erythropoietin is the principal mediator of stress erythropoiesis. The requirement for Epo receptor (EpoR) signaling appears to be even greater during stress than during basal erythropoiesis (Liu et al., 2006). Mice expressing reduced EpoR numbers (Jegalian et al., 2002) or lacking cytoplasmic tyrosines (Zang et al., 2001) have normal basal erythropoiesis but a deficient stress response.

Some forms of anemia demonstrate a relatively weak response to elevated Epo levels rather than the vigorous response characterizing stress erythropoiesis. These include certain physiologic states such as pregnancy and the early anemia of infancy, as well as pathologic states such as the anemia of inflammation (AI; also called the anemia of chronic disease, ACD), chronic kidney disease and iron deficiency anemia (IDA). In each of these settings, the erythropoietic response is enhanced to varying degrees by the provision of additional iron. Conversely, iron restriction has a suppressive effect on erythropoiesis that is characterized by a blunted response to Epo in *ex vivo* systems, animal studies, and human subjects (Hillman and Henderson, 1969; Kimura et al., 1986; Goodnough, 2007; Tanno et al., 2008). The relevance of Tf-bound iron to stress erythropoiesis is demonstrated by the defective response in aceruloplasminemic mice (Cherukuri et al., 2004).

Iron is delivered to the erythron for heme production by the glycoprotein transferrin (Tf) and its interaction with the classic transferrin receptor (Tfr1). Tf functions not only as a cargo, but also a signaling molecule. Mounting evidence suggests that Tf communicates circulating iron status to hepatocytes and erythroblasts (Lin et al., 2007; Bartnikas et al., 2011; Parrow et al., 2019) by interactions with transferrin receptor 2 (TfR2) (Forejtnikovà et al., 2010) (Bullock et al., 2010; Khalil et al., 2018). The mechanisms by which this signaling participates in cross communication between iron metabolism and erythropoiesis are unknown.

The setting of IDA provides an opportunity to investigate the relationship between iron status and erythropoiesis in the absence of other pathologic confounders. Dietary iron deficiency produces sequential changes: depletion of hepatic and splenic stores, decrease in circulating iron, decreased iron incorporation into hemoglobin, fall in blood oxygen carrying capacity, upregulation of Epo. The response of the erythron to the elevated Epo is blunted until iron is provided (Jansson et al., 1985). Upon iron repletion, recovery from IDA has features of stress erythropoiesis, including rapid erythropoietic expansion and reticulocytosis. Splenomegaly is evidenced in mice (Gibson et al., 2011) and patients (Wei et al., 2021) with severe ID, consistent with extramedullary hematopoiesis.

We present here studies which characterize the iron status, hematologic parameters, and expression of key signaling molecules in mice with dietary iron deficiency with or without anemia. We moreover assess the acute responses to the administration of iron-loaded Tf. Our observations support the importance of iron-loaded Tf in modulating the erythropoietic response in iron deficiency anemia.

## Methods

### Mouse Models

Wild type FVB/n mice were bred and maintained in the animal facility at Saint Louis University under standard conditions with 12-h light cycles and *ad libitum* access to food and water. Defined iron diets were used to generate murine models of iron deficiency with or without anemia. Control mice, were weaned and maintained on a standard chow diet containing 226 ppm iron (Teklad Global 2018S). To induce iron deficiency, pups were weaned onto a purified chow containing 67 ppm iron (TestLab TD 5755) until they were 4 weeks of age and were then fed identical chow but containing <2 ppm (TestLab TD 5859) added iron (with 10–20 ppm residual iron) until sacrifice. To induce iron deficiency anemia, dams and pups were fed the TD 5859 iron diet. In indicated experiments, 10 mg of human holotransferrin (Sigma Aldrich, St. Louis, MO) or carrier was delivered by intraperitoneal injection. Mice were sacrificed 6 h after injection. All analyses were performed at 5–6 weeks of age. Animal studies were performed under Institutional Animal Care and Use Committee approved protocols.

### Hematologic and Serum Parameters

Complete blood counts were assayed on a Cell-Dyn 3,700 analyzer (Advanced Veterinary Laboratories, St Louis, MO). Serum iron concentrations and unsaturated iron binding capacity (UIBC) were assayed per manufacturer’s protocol (Pointe Scientific, Canton, MI). Total iron binding capacity (TIBC) was calculated as the sum of serum iron plus UIBC and Tf saturation was calculated as serum iron divided by TIBC. Serum erythropoietin (Epo) was measured by enzyme-linked immunosorbent assay (R&D Systems, Minneapolis, MN).

### Tissue Iron Concentrations

Liver and spleen specimens were analyzed for non-heme iron content by the bathophenanthroline method of Torrance and Bothwell (Torrance et al., 1980) as previously described (Fleming et al., 2001).

### Quantitative Reverse-Transcription Polymerase Chain Reaction

Tissue samples were homogenized in guanidium thiocyanate (Trizol, Carlsbad, CA). RNA was extracted per manufacturer’s recommendation and reverse transcribed with iScript RT Supermix (Biorad, Hercules, CA). RT-PCR was performed on a CFX Connect real time system (Biorad) with Taqman gene expression primers and probes (Applied Biosystems, Foster City, CA). Relative gene expression was analyzed by the ΔCt method (Livak and Schmittgen, 2001).

### Statistical Analyses

Statistical analyses were performed with GraphPad Prism. Where appropriate, outliers identified by the Rout method were removed. Data were analyzed by analysis of variance (ANOVA) with Sidak’s multiple comparison test of selected groups (as identified in the figures). *p*-values < 0.05 considered statistically significant Student’s *t* test.

## Results

**FIGURE 1:**
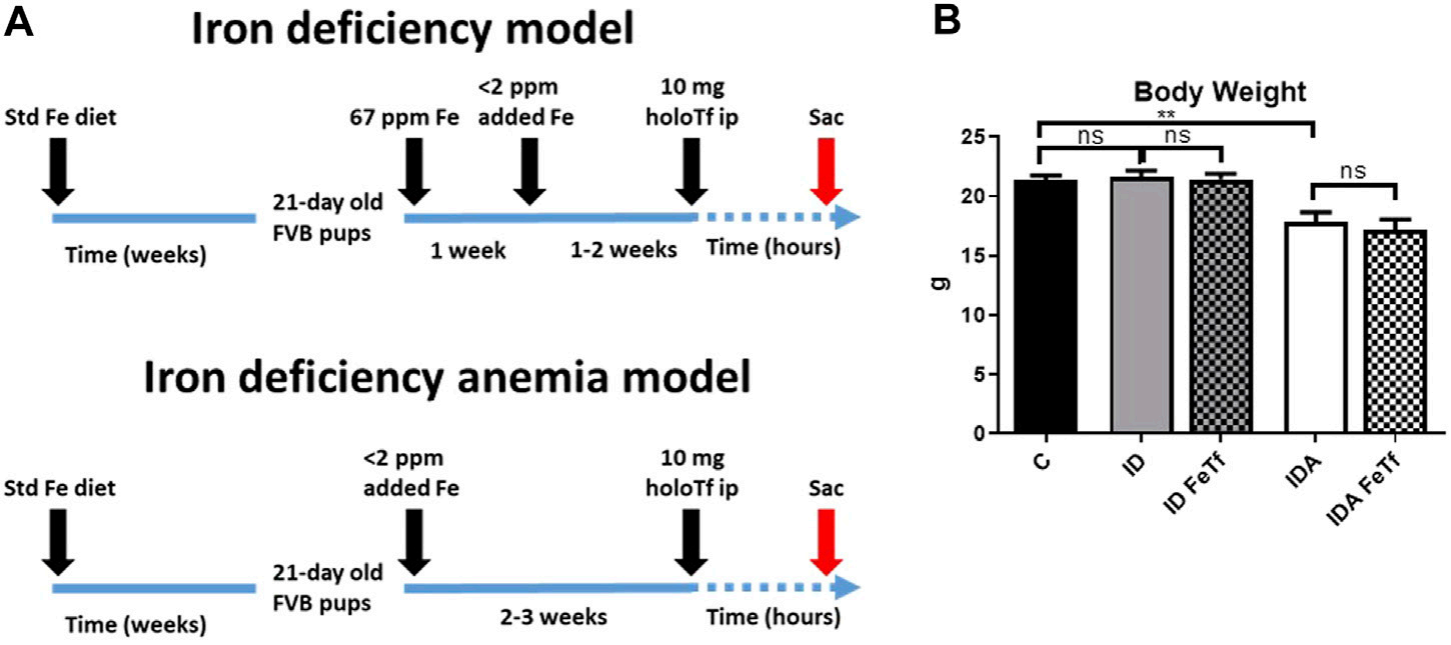
Dietary regimens produce murine models of iron deficiency and iron deficiency anemia. **(A)** A schematic of the dietary regimens used for each model is shown. Iron deficient mice (ID) were generated by weaning 21-day old mice onto 60 ppm Fe diets until 4 weeks of age then 2 ppm Fe diets until sacrifice. A murine model of iron deficiency anemia (IDA) was generated by feeding mice a 2 ppm Fe diet. At 5–6 weeks of age, both murine models were given 10 mg human holotransferrin (or carrier) by intraperitoneal injection and sacrificed for analysis 6 h post-injection. **(B)** Body weights were measured. Control mice fed a standard 250 ppm Fe diet are included for reference. For control mice *n* = 25, for ID cohorts *n* = 16–20 and for IDA cohorts, *n* = 6-7 mice per group. ppm, parts per million. ppm, parts per million; data represented as mean (SEM).*, *p* < 0.05; ***, *p* < 0.005; ****, *p* < 0.001; ns, non-significant.

**FIGURE 2:**
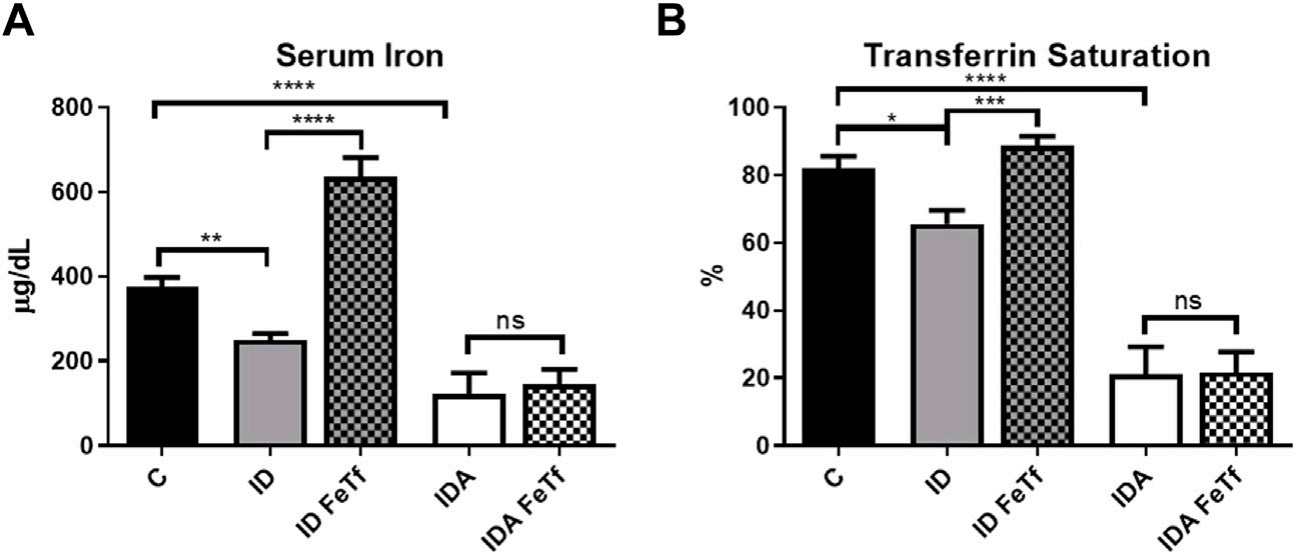
Acute holotransferrin administration reverses the decrease in serum iron parameters in mice with ID but not IDA. ID and IDA mice were treated with carrier or holotransferrin and **(A)** serum iron and **(B)** transferrin saturation were measured. Control mice fed a standard 250 ppm Fe diet are included for reference. For control mice, *n* = 8, for ID cohorts *n* = 13–15 and for IDA cohorts, *n* = 6 mice per group. ppm, parts per million; data represented as mean (SEM).*, *p* < 0.05; ***, *p* < 0.005; ****, *p* < 0.001; ns, non-significant.

**FIGURE 3:**
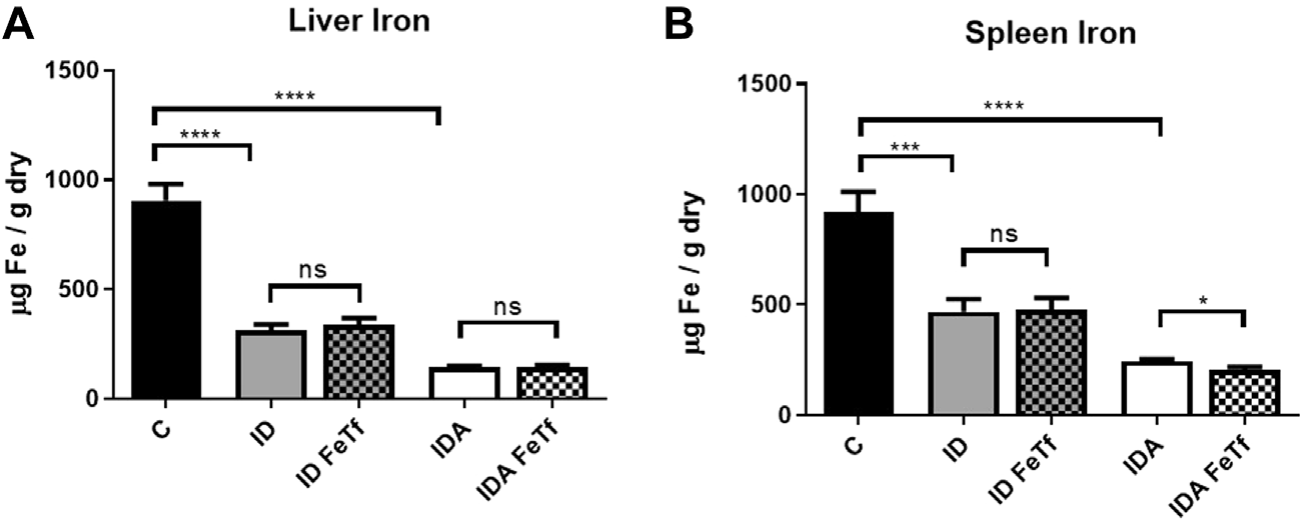
Tissue iron progressively decreases with severity of iron restriction in mice with ID and IDA. ID and IDA mice were treated with carrier or holotransferrin. **(A)** Liver iron concentrations and **(B)** spleen iron concentrations were measured. Control mice fed a standard 250 ppm Fe diet are included for reference. For control mice, *n* = 5-6, for ID cohorts *n* = 15–20 and for IDA cohorts *n* = 5-7 mice per group. ppm, parts per million; data represented as mean (SEM).*, *p* < 0.05; ***, *p* < 0.005; ****, *p* < 0.001; ns, non-significant.

**FIGURE 4:**
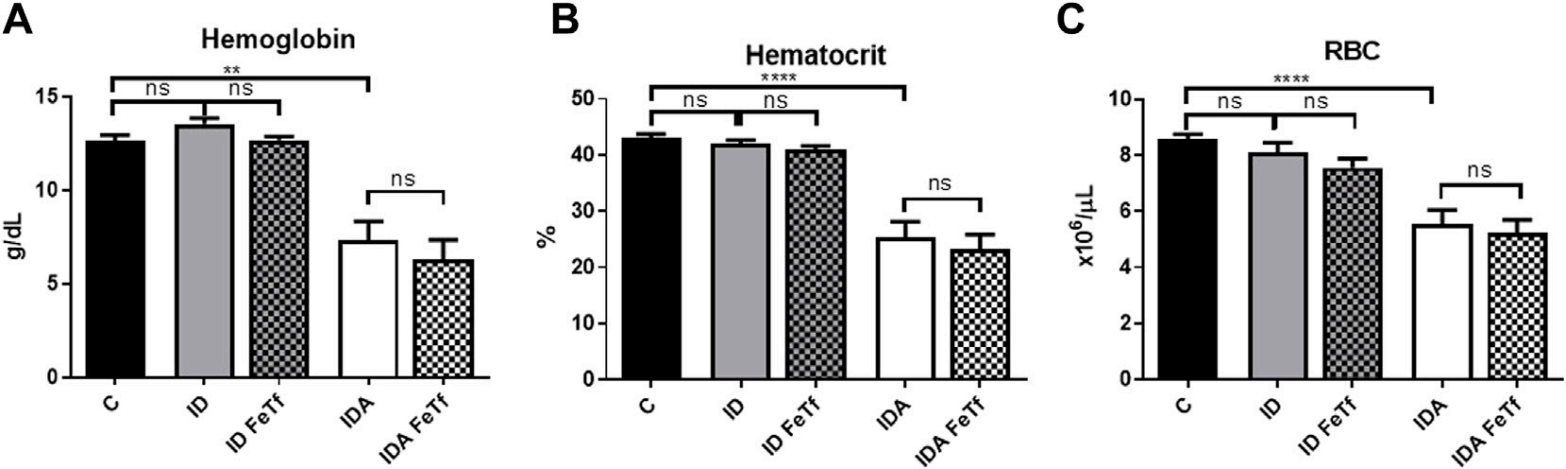
Acute holotransferrin administration does not alter the erythroid phenotypes found in the murine models of ID and IDA. ID and IDA mice were treated with carrier or holotransferrin. **(A)** Hemoglobin, **(B)** hematocrit and **(C)** red blood cell counts were measured. Control mice fed a standard 250 ppm Fe diet are included for reference. For control mice *n* = 16, for ID cohorts *n* = 6–11 and for IDA cohorts *n* = 6-7 mice per group. ppm, parts per million; Data represented as mean (SEM) *, *p* < 0.05; ***, *p* < 0.005; ****, *p* < 0.001; ns, non-significant.

**FIGURE 5:**
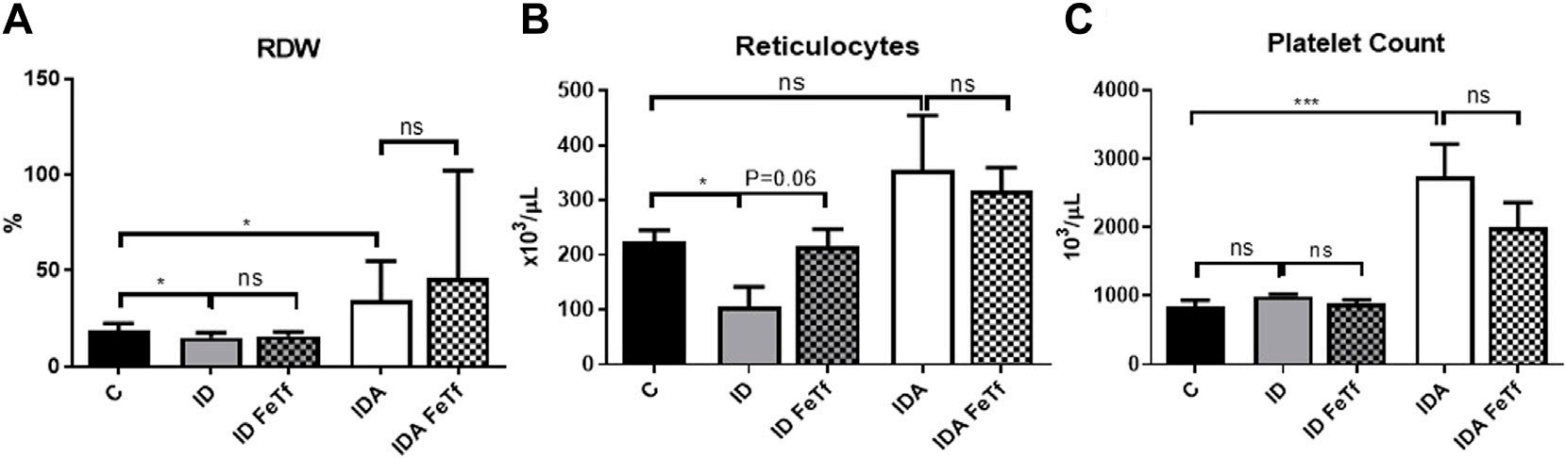
Mice with iron deficiency anemia demonstrate suppressed erythropoietic activity and enhanced thrombopoiesis. ID and IDA mice were treated with carrier or holotransferrin. **(A)** Red cell distribution width, **(B)** reticulocyte counts and **(C)** platelet counts were measured. Control mice fed a standard 250 ppm Fe diet are included for reference. For control mice *n* = 16, for ID cohorts *n* = 6–11 and for IDA cohorts *n* = 6-7 mice per group. ppm, parts per million; data represented as mean (SEM).*, *p* < 0.05; ***, *p* < 0.005; ****, *p* < 0.001; ns, non-significant.

Iron and hematologic parameters in murine models of iron deficiency and iron deficiency anemia. We utilized a mouse strain (FVB/n) with inherently high iron status, and placed them on diets with lower than standard dietary iron concentrations to empirically identify regimens generating iron deficiency without or with anemia. The successful regimens required initiation of low iron diets early in life, during the phase of rapid growth and expansion of the blood mass. A successful regimen (Figure 1A) separated mice into those defined as iron deficiency only (ID), and those defined as iron deficiency with anemia (IDA). The ID mice had lower circulating iron (Figure 2) and tissue iron stores (Figure 3) compared with control mice, without differences in blood hemoglobin, hematocrit, or RBC values (Figure 4). The regimen defined as IDA produced mice with more profound iron deficiency and consequent anemia. These IDA mice had substantially lower serum iron concentration and transferrin saturations (Figure 2) and tissue iron stores (Figure 3) than the ID mice. The greater decrement in transferrin saturation than serum iron in the IDA mice reflects the expected greater total iron binding capacity in IDA. The mice were anemic, with blood hemoglobin concentrations, hematocrits, and RBC counts significantly lower in the IDA mice compared with the ID mice (Figure 4). These mice also demonstrated the expected increase in red blood cell distribution width (RDW, Figure 5A). As expected, the RBC mean corpuscular volume was decreased in the IDA mice and the mean corpuscular hemoglobin concentrations were unaffected (not shown). The IDA anemia affected the somatic growth of these mice, and they had lower body weights than control mice or mice with ID alone (Figure 1B).

**FIGURE 6:**
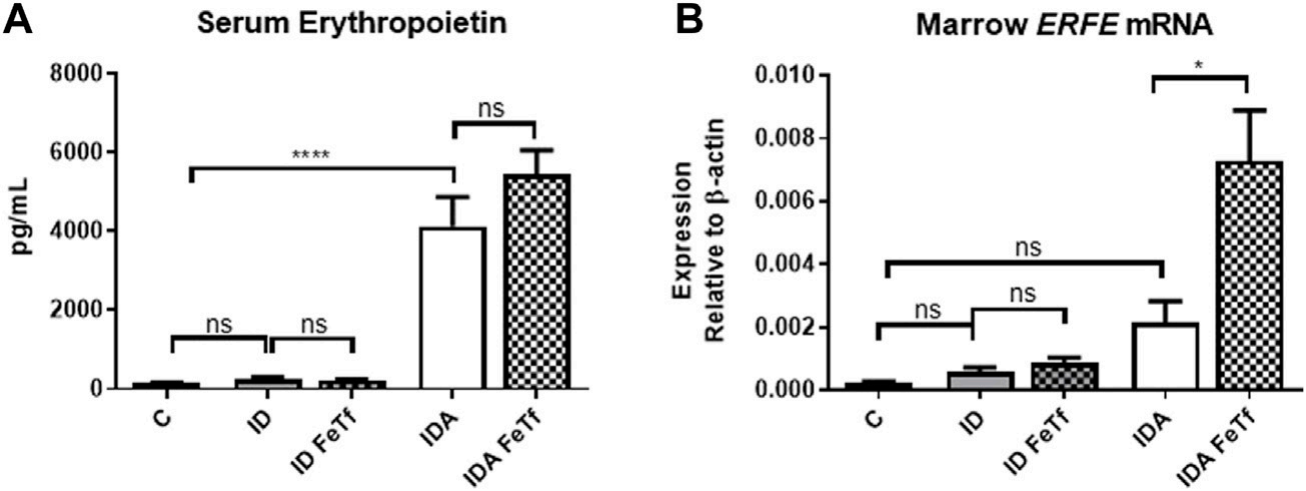
Exogenous transferrin enhances erythropoietin-mediated upregulation of erythroferrone in mice with iron deficiency anemia. ID and IDA mice were treated with carrier or holotransferrin. **(A)** Serum erythropoietin and **(B)** marrow *Erfe* mRNA were measured. Serum erythropoietin: for control mice *n* = 5, for ID cohorts *n* = 2-5 and for IDA cohorts *n* = 5 mice per group. Marrow *Erfe* mRNA: for control mice *n* = 3, for ID cohorts *n* = 13 and for IDA cohorts *n* = 6-7 mice per group. Control mice fed a standard 250 ppm Fe diet are included for reference. ppm, parts per million; data represented as mean (SEM).*, *p* < 0.05; ***, *p* < 0.005; ****, *p* < 0.001; ns, non-significant.

IDA mice had evidence of relative erythroid hypoproliferation. Whereas erythropoietin (Epo) concentrations were substantially elevated in the IDA mice (Figure 6A), they had a very modest (and not statistically significant) increase in reticulocyte count (Figure 5B) and decreased number of circulating RBCs (Figure 4C). By contrast, thrombopoiesis was enhanced, as reflected in a markedly increased platelet count (Figure 5C). These features of erythroid suppression were not observed in the ID mice. There was a lower reticulocyte count in the ID compared with control mice (Figure 5B) despite similar serum Epo concentrations (Figure 6A), suggesting a minimal effect on erythropoiesis by iron deficiency alone.

**FIGURE 7:**
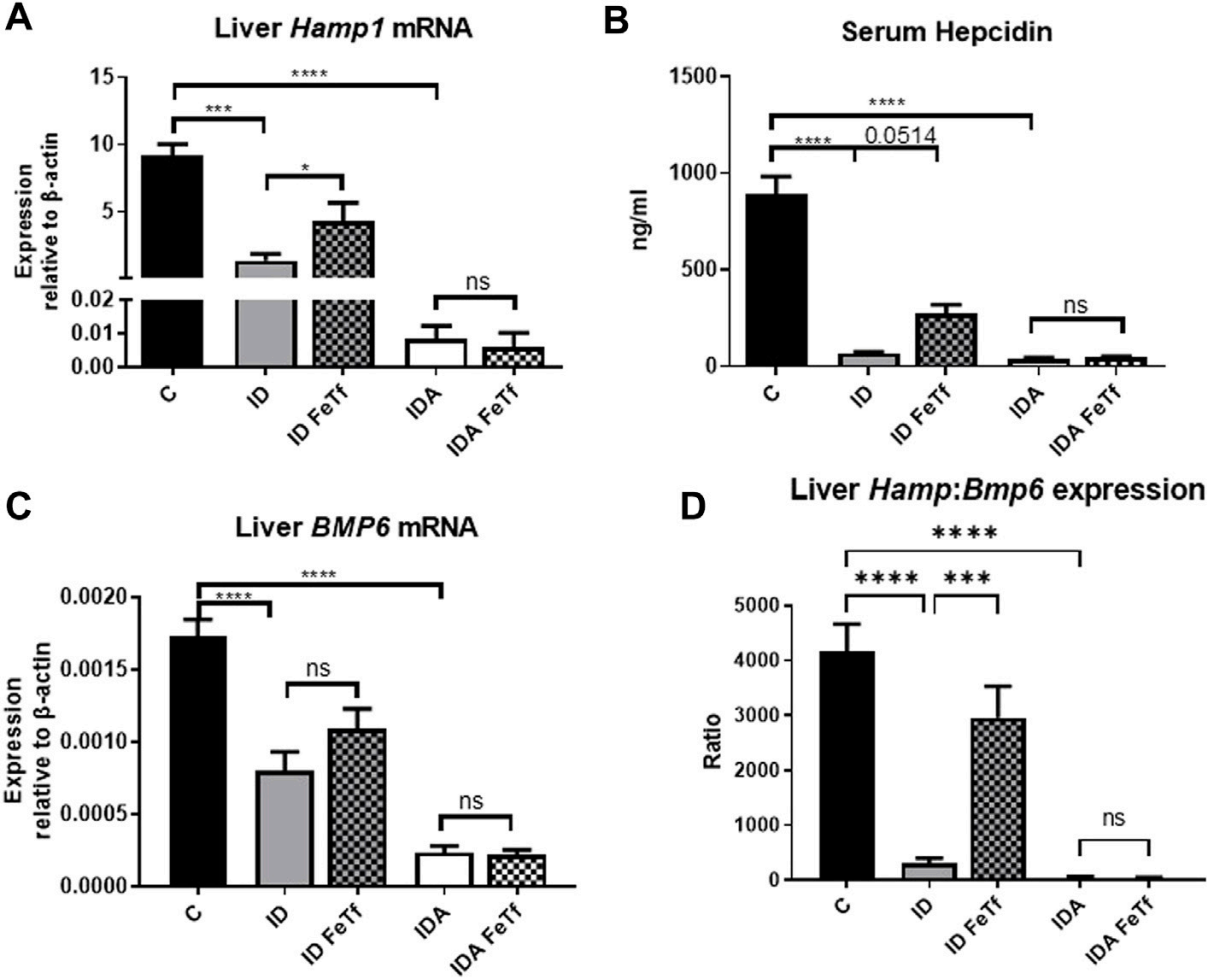
Exogenous transferrin increases hepatic hepcidin expression in mice with iron deficiency. ID and IDA mice were treated with carrier or holotransferrin. **(A)** Liver *Hamp1* mRNA, **(B)** serum hepcidin and **(C)** liver *Bmp6* mRNA were measured. **(D)** Liver *Hamp1* mRNA was indexed to liver *Bmp6* mRNA. qPCR assays: control mice *n* = 10–13, for ID cohorts *n* = 7–14 and for IDA cohorts *n* = 6-7 mice per group. Serum assay: for control mice *n* = 10, for ID cohorts *n* = 3 and for IDA cohorts *n* = 3-4 mice per group. Control mice fed a standard 250 ppm Fe diet are included for reference. ppm, parts per million; data represented as mean (SEM).*, *p* < 0.05; ***, *p* < 0.005; ****, *p* < 0.001; ns, non-significant.

Iron deficiency down-regulates *Hamp1* relative to *Bmp6* independent of serum Epo or marrow *Erfe*. We examined relationships between iron status and signaling events regulating hepcidin expression in the ID and IDA mouse models. As expected, liver *Hamp1* (Figure 7A) and circulating hepcidin concentrations (Figure 7B) were lower in the ID mice, and further decreased in the mice with IDA. The decreases in *Hamp1* expression were associated with decreases in known iron signals, i.e., liver iron concentration (Figure 3A) and serum transferrin saturation (Figure 2B). *Hamp1* downregulation was not dependent upon upregulation of erythroid hepcidin suppressor erythroferrone, as marrow expression of the *Erfe* (aka *Fam132b*) gene was unaffected in the ID mice. As seen with *Hamp1*, *Bmp6* expression was lower in the ID compared with control mice, and further decreased in the IDA mice (Figure 7C). However, the magnitude of the effect of iron deficiency on these genes is dissimilar, such that *Hamp1* indexed to *Bmp6* is markedly less in the ID mice compared to control, and decreased further in the IDA mice (Figure 7D). This observation is consistent with suppression of BMP6 signaling in iron deficiency.

Holotransferrin administration increases liver *Hamp1* relative to *Bmp6* expression in ID but not IDA mice. To aid in distinguishing the role of transferrin saturation from liver iron concentration in the regulation of hepcidin in these model systems, we examined the acute effect of exogenous administration of holotransferrin (holoTf). Changes in iron parameters have previously been reported in mice 6 h after holoTf administration, suggesting that it would provide an appropriate timeframe for these investigations (Ramos et al., 2011) Administration of holoTf was reflected in an increase in serum iron and Tf saturation in mice with ID. Holotransferrin administration raised the serum iron in these mice, and increased the transferrin saturation. Administration of holoTf had no effect on iron parameters in the mice with IDA, presumably due to utilization of the administered iron within the 6 h timeframe by the erythron and iron deficient tissues in this setting. In the ID mice, holoTf administration significantly increased liver *Hamp1* expression (Figure 7A) without an increase in liver iron concentration. However, no effect of holoTf administration on *Hamp1* expression was observed in the IDA mice. Serum hepcidin concentrations showed a similar profile (Figure 7B), as did expression of another BMP-SMAD target gene *Atoh8* (not shown). Acute holoTf administration had only a modest (and not statistically significant) effect on liver *Bmp6* expression in the ID mice, and no effect in the IDA mice (Figure 7C). Liver *Hamp1* indexed to *Bmp6* demonstrated a marked increase following treatment with holoTf in mice with ID, but not mice with IDA (Figure 7D). We speculate that in the setting of iron deficiency an increase in the concentration of circulating holoTf, relative to unsaturated Tf form(s), enhances hepatocellular BMP6 sensitivity.

Holotransferrin administration increases marrow *Erfe* expression relative to serum Epo in mice with IDA (Figure 6A). To determine the basis for the lack of liver *Hamp1* upregulation in response to holoTf in the IDA mice, we examined the effects holoTf on the erythroid system and expression of the erythroid regulator Erfe. As expected, exposure to holoTf did not acutely (i.e., at 6 h) influence circulating blood cell parameters. No significant effect was observed in serum Epo concentrations. Nonetheless holoTf administration led to a striking increase in expression of marrow *Erfe/Fam132b* (Figure 6B). As *Erfe* is an Epo-responsive gene (Kautz et al., 2015), these findings suggest that holoTf increases erythroid Epo sensitivity. The absence of *Hamp1* upregulation by holoTf in the IDA mice can be plausibly attributed to enhanced Epo-mediated upregulation of *Erfe* and its suppressive effect on BMP signaling*.*

## Discussion

This study was undertaken to characterize the participation of Tf as a signaling molecule regulating iron homeostasis and erythropoiesis. The mechanism by which Tf signals iron status to regulate hepcidin has not been fully elucidated, in part because of the rapid dedifferentiation of primary hepatocytes in culture (Bartnikas et al., 2011), and thus requiring *in vivo* systems. Such understanding is relevant to the means by which erythron iron demand and plasma iron availability are communicated. These events are of particular importance in settings of stress erythropoiesis, including recovery from iron deficiency anemia. We therefore generated mouse models for two states in which cross communication between the liver and erythron require modulation: iron deficiency, and iron deficiency anemia. In iron deficiency without anemia, hematological parameters are maintained by the depletion of stored iron, in association with decreased hepcidin. Our observations confirm that in this setting, Tf provides a major signal regulating hepcidin expression. This is demonstrated by the ability of iron-loaded Tf to upregulate hepcidin in the absence of changes in other regulatory signals (i.e., liver iron concentration, Erfe). Our studies moreover demonstrate that this regulation occurs by affecting the relationship between liver Bmp6 and hepcidin expression. The *Hamp1:Bmp6* ratio is suppressed in mice with ID compared to control mice, but is largely normalized by the administration of holoTf. This occurs in an acute (<6 h) timeframe. We speculate that this arises from a sensitization of the hepatocyte to BMP paracrine signaling. Potential mechanisms include changes in the functional activity of matriptase2 (and thus the BMP co-receptor hemojuvelin) or changes in the surface expression of the BMP receptor complex. Regardless, it is likely the regulation of hepcidin involves the interaction of Tf with hepatocellular transferrin receptor 2 (TfR2), with downstream effects on SMAD1,5,8 and/or MAPK phosphorylation (Bartnikas et al., 2011) (Ramey et al., 2009) (Lin et al., 2007). Some evidence supports a direct interaction between the extracellular domain of TfR2 and certain BMPs that is influenced by the presence of holoTf (Rauner et al., 2019).

Our studies do not support a significant role for erythroid-related signaling in the regulation of hepcidin in ID without anemia. It has been suggested that the activity of prolyl hydroxylases participating in Epo degradation might be affected by not only hypoxia but also iron availability (Kaelin and Ratcliffe, 2008). We observed that serum Epo levels and Erfe expression are unaffected by the magnitude of iron deficiency observed in our ID mice. Our studies are unable to determine if the increase in Epo seen in the IDA mice is entirely due to anemia/hypoxia or also due to a lower iron status. We did observe an increase in reticulocyte count after FeTf administration in the ID mice. This increase over the short timeframe of these experiments may reflect a shortened marrow transit time, as reported in iron-deficient rats (Tarbutt, 1969).

In the setting of iron deficiency anemia, Erfe appears to be the dominant regulator of hepcidin expression. We observed that the *Hamp:Bmp6* ratio is suppressed even further in the IDA mice compared with ID mice, consistent with evidence that Erfe inhibits signaling to hepcidin by sequestering bone morphogenetic proteins (Arezes et al., 2018; Wang et al., 2020). In IDA, exogenous holoTf fails to increase hepcidin. There was instead an effect of holoTf on marrow *Erfe* expression, with a further increase in *Erfe* expression. This occurred without upregulation of serum Epo, suggesting that the increased *Erfe* is a manifestation of increased erythroid Epo sensitivity.

We have previously demonstrated that Tf is capable of modulating Epo sensitivity (Parrow et al., 2019), and others have demonstrated that TfR2 also plays a role (Nai et al., 2015) (Wallace et al., 2015). Erythroid TfR2 is a component of the Epo receptor complex (Forejtnikovà et al., 2010). Moreover, a role for TfR2 has been identified in mediating the attenuated Epo sensitivity that characterizes the marrow iron-restriction response (Bullock et al., 2010; Khalil et al., 2018). Surface expression of TfR2 is downregulated in iron deficiency (Khalil et al., 2018). Plausibly, Epo receptor expression or activity is influenced by the presence of cell surface TfR2. Studies in the human erythroid cell line UT-7/Epo (Fouquet et al., 2021) demonstrated stabilization of EpoR by Fe-Tf in cells expressing TfR2, but no such effect in cells without TfR2. Paradoxically, however, cells without TfR2 had more EpoR with or without Fe-Tf, and greater Epo-responsiveness. Perhaps TfR2 can act to enhance or suppress Epo signaling dependent upon the relative distribution of different Tf forms. Additionally, TfR1 may play a role in mediating certain effects of Fe-Tf on Epo responsiveness (Coulon et al., 2011; Fouquet et al., 2021).

The data presented here demonstrate that administration of exogenous Tf conveys or enhances an iron signal to hepcidin, resulting in its upregulation in mice with ID. In contrast, under conditions of IDA exogenous Tf exerts it effects on hepcidin by enhancing the erythroid signal, maintaining suppression of hepcidin to allow for continued uptake of iron until anemia has improved. Elucidation of the precise mechanisms by which this occurs and the relative contributions of TfR1 and TfR2 to these processes are important avenues of future investigation.

## Data Availability

The raw data supporting the conclusion of this article will be made available by the authors, without undue reservation.
